# Loss of Maternal ATRX Results in Centromere Instability and Aneuploidy in the Mammalian Oocyte and Pre-Implantation Embryo

**DOI:** 10.1371/journal.pgen.1001137

**Published:** 2010-09-23

**Authors:** Claudia Baumann, Maria M. Viveiros, Rabindranath De La Fuente

**Affiliations:** 1 Female Germ Cell Biology Group, Department of Clinical Studies, University of Pennsylvania, Kennett Square, Pennsylvania, United States of America; 2 Department of Animal Biology, Center for Animal Transgenesis and Germ Cell Research, School of Veterinary Medicine, University of Pennsylvania, Kennett Square, Pennsylvania, United States of America; Massachusetts General Hospital, Howard Hughes Medical Institute, United States of America

## Abstract

The α-thalassemia/mental retardation X-linked protein (ATRX) is a chromatin-remodeling factor known to regulate DNA methylation at repetitive sequences of the human genome. We have previously demonstrated that ATRX binds to pericentric heterochromatin domains in mouse oocytes at the metaphase II stage where it is involved in mediating chromosome alignment at the meiotic spindle. However, the role of ATRX in the functional differentiation of chromatin structure during meiosis is not known. To test ATRX function in the germ line, we developed an oocyte-specific transgenic RNAi knockdown mouse model. Our results demonstrate that ATRX is required for heterochromatin formation and maintenance of chromosome stability during meiosis. During prophase I arrest, ATRX is necessary to recruit the transcriptional regulator DAXX (death domain associated protein) to pericentric heterochromatin. At the metaphase II stage, transgenic ATRX-RNAi oocytes exhibit abnormal chromosome morphology associated with reduced phosphorylation of histone 3 at serine 10 as well as chromosome segregation defects leading to aneuploidy and severely reduced fertility. Notably, a large proportion of ATRX-depleted oocytes and 1-cell stage embryos exhibit chromosome fragments and centromeric DNA–containing micronuclei. Our results provide novel evidence indicating that ATRX is required for centromere stability and the epigenetic control of heterochromatin function during meiosis and the transition to the first mitosis.

## Author Summary

The transmission of an abnormal chromosome complement from the gametes to the early embryo, a condition called aneuploidy, is a major cause of congenital birth defects and pregnancy loss. Human embryos are particularly susceptible to aneuploidy, which in the majority of cases is the result of abnormal meiosis in the female gamete. However, the molecular mechanisms involved in the onset of aneuploidy in mammalian oocytes are not fully understood. We show here that, the α-thalassemia/mental retardation X-linked protein (ATRX) is essential for the maintenance of chromosome stability during female meiosis. ATRX is required to recruit the transcriptional regulator DAXX to pericentric heterochromatin at prophase I of meiosis. Notably, lack of ATRX function at the metaphase II stage interferes with the establishment of chromatin modifications associated with chromosome condensation leading to segregation defects, chromosome fragmentation, and severely reduced fertility. Our results provide direct evidence for a role of ATRX in the regulation of pericentric heterochromatin structure and function in mammalian oocytes and have important implications for our understanding of the epigenetic factors contributing to the onset of aneuploidy in the female gamete.

## Introduction

Heterochromatin formation in eukaryotic cells is essential for the maintenance of nuclear architecture, the control of gene expression and chromosome segregation [1]–[6]. In the mouse genome, constitutive heterochromatin consists of two closely related chromosomal sub-domains with distinct structure and function [7], [8]. Centric heterochromatin, is epigenetically determined by deposition of the histone variant CENP-A (centromere associated protein-A), contains several hundred kilobases of the 120 bp repeat unit of the minor satellite sequence and regulates the assembly of a single kinetochore on each sister chromatid required for microtubule attachment [9], [10]. In contrast, pericentric heterochromatin comprises several megabases of the 234 bp repeat of the major satellite sequence and is marked by transcriptionally repressive histone modifications such as trimethylation of lysine 9 on histone H3 as well as chromatin remodeling proteins such as heterochromatin protein 1 (HP1) and members of the SWI/SNF2 (switch/sucrose non-fermenting 2) family including ATRX [7], [10]–[12].

In several organisms including mammals, pericentric heterochromatin formation is essential to coordinate sister centromere cohesion and for the timely separation of individual chromatids during mitosis [2], [3], [7], [10]. Importantly, members of the SWI/SNF2 protein family specifically recruited to pericentric heterochromatin are essential to maintain sister chromatid cohesion until the onset of anaphase in order to ensure accurate chromosome segregation. For example, chromatin-remodeling complexes such as SNF2h are essential to load the cohesin subunit RAD21 at the centromeres of human mitotic cells [13]. In addition, loss of HP1 from pericentric heterochromatin in mouse somatic cells deficient for the histone methyltransferase SUV39H1/2 disrupts centromeric cohesion [14], [15]. ATRX associates with constitutive heterochromatin domains in human and mouse somatic cells, as well as with facultative heterochromatin of the inactive X chromosome in mouse somatic and trophoblast stem cells, suggesting a potential role in heterochromatin formation [11], [12], [16]–[18]. Therefore, while a growing body of evidence indicates that pericentric heterochromatin formation may have a significant impact on centromere cohesion during mitosis, little is known about this critical process during female mammalian meiosis.

The centromeres of meiotic chromosomes exhibit unique structural and functional properties that are essential to coordinate dynamic molecular interactions with the microtubular network and ensure accurate chromosome segregation. For instance, cohesion along sister chromatids and at centromeres during metaphase I is required to coordinate homologous chromosome segregation whereas maintenance of sister centromere cohesion at the metaphase II stage is required for the timely separation of sister chromatids during anaphase II onset [19], [20]. We have previously demonstrated that ATRX binds to pericentric heterochromatin in fully-grown mouse oocytes and that its functional ablation *in vitro* severely disrupts chromosome alignment at the metaphase II (MII) spindle [12]. Consistent with these studies, transient depletion of ATRX protein following siRNA transfection in HeLa cells resulted in chromosome congression defects during mitosis [18]. The abnormal chromosome alignment observed in mouse oocytes at the metaphase II stage [12] suggests that loss of ATRX function might interfere with proper chromosome segregation during meiosis and result in the potential transmission of aneuploidy to the developing conceptus. However, the molecular mechanisms of ATRX function during meiosis as well as the functional consequences of maternal ATRX ablation on chromosome stability in the early pre-implantation embryo remained to be determined. Here, we induced an oocyte-specific knockdown of the ATRX protein using a transgenic RNAi approach [21]–[23]. Our results indicate that ATRX plays a critical role in the functional differentiation of chromatin structure during oogenesis and underscore the importance of chromatin remodeling proteins in the control of chromosome segregation during meiosis.

## Results

### Oocyte-specific knockdown of ATRX in a transgenic RNAi mouse model

Following pronuclear microinjection, a total of seven founder mice (3 females and 4 males) were obtained showing successful transgene integration. Analysis of the F1 progeny indicated that three male founders (#A-6, #B-23 and #C-11) and two female founders (#G-5 and #H-10) efficiently transmitted the transgene to their offspring (Figure S1B). Microscopic examination of histological sections of gonadotropin stimulated and non-stimulated ovaries from control littermates and transgenic females revealed the presence of oocytes at different stages of growth and follicular maturation, indicating that ATRX ablation does not adversely affect oocyte growth (Figure S1C).

**Figure 1:**
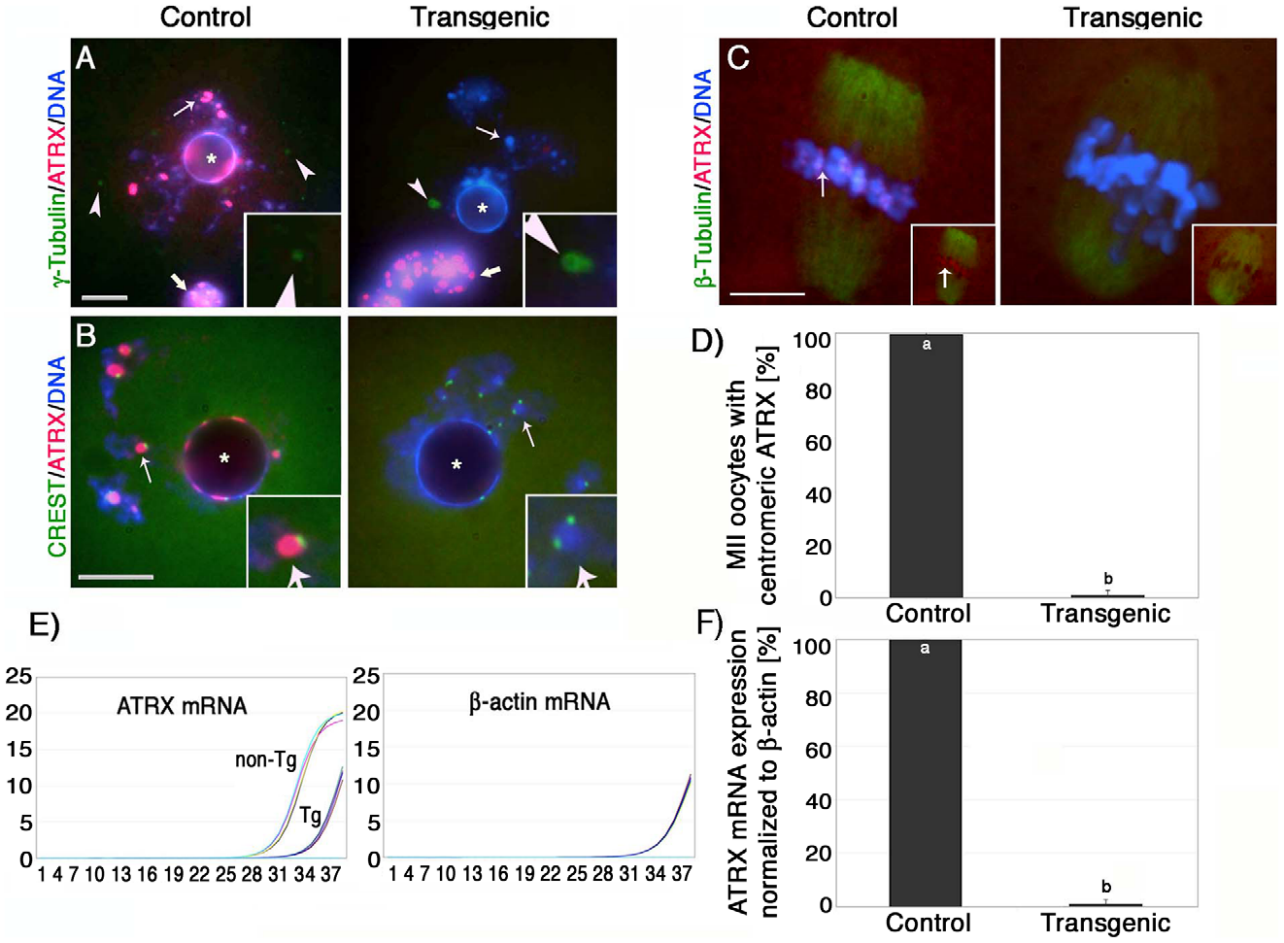
Oocyte-specific ablation of the ATRX protein in a transgenic RNAi mouse model. (**A**) Nucleus of control oocyte showing bright ATRX staining (red, left image) at heterochromatin domains (arrow) as well as γ-tubulin signals (green) towards the nuclear periphery (arrowheads and inset). No ATRX staining is detectable in the nucleus of transgenic oocytes (arrow, right image). However, γ -tubulin staining remained unaffected (arrowheads and inset). Note that ATRX protein is expressed in surrounding somatic granulosa cells in both control and transgenic animals (bold arrows). The position of the nucleolus is indicated by (*). (**B**) Partial co-localization of ATRX and CREST signals at pericentric heterochromatin domains in control oocytes (arrow and inset, left image). By contrast, while localization of CREST signals is unaffected in transgenic oocytes (arrow and inset, right image), ATRX is not detectable at DAPI-bright heterochromatin domains. (**C**) Control MII oocyte immunostained with ATRX (red, arrow) and β-tubulin (green) showing proper chromosome alignment at the metaphase II spindle (left image, arrow, inset shows ATRX/β-tubulin merge only). Lack of ATRX in transgenic oocytes (right image, inset) results in abnormal chromosome alignment. (**D**) Proportion of *in vivo* matured MII stage oocytes that exhibited ATRX at centromeric heterochromatin. (**E–F**) Quantitative analysis of *Atrx* transcripts by real-time PCR revealed a significant reduction (p<0.001) in the levels of *Atrx* mRNA in transgenic (Tg) oocytes compared with controls (non-Tg). Analysis of β-actin mRNA was used as a housekeeping control. All data are presented as the mean ± s.d. of three independent experiments. Scale bars  = 10 µm.

Consistent with our previous experiments [12], ATRX (red) was found associated with bright DAPI stained heterochromatin foci as well as with the peri-nucleolar heterochromatin rim in control pre-ovulatory oocytes at the germinal vesicle stage where it partially co-localized with kinetochores detected by CREST (calcynosis Raynaud's phenomenon esophageal dismotility, sclerodactyly and telangiectasia) (Figure 1A and 1B). Moreover, ATRX was also present at heterochromatin foci in surrounding somatic granulosa cells (Figure 1A; thick arrow). In contrast, ATRX was undetectable at the germinal vesicle in >95% of transgenic oocytes of line #G-5 (Figure S1D) in which only somatic granulosa cells exhibited bright ATRX staining therefore confirming the oocyte-specific ablation of ATRX in our transgenic mouse model (Figure 1A). The cytoplasmic signals for γ-tubulin staining (green) located near the germinal vesicle of transgenic oocytes remained unaffected, further attesting for the specificity of this approach (Figure 1A; inset). Importantly, analysis of CREST signals in transgenic oocytes revealed that the kinetochore-associated proteins detected by this antiserum remain unaffected in the absence of ATRX function (Figure 1B; inset).

At the metaphase II stage, ATRX exhibits a prominent localization to the centromeres of meiotic chromosomes in 99.4% of control oocytes (n = 86). In contrast, the proportion of transgenic oocytes (0.9%; n = 93) with ATRX staining at pericentric heterochromatin in line #G-5 was significantly reduced (P<0.001) indicating that expression of the *Atrx*-hairpin caused an effective reduction of ATRX protein levels in the majority of mature oocytes (Figure 1C and 1D, insets). Moreover, comparison of mRNA expression in pools of transgenic and control metaphase II stage oocytes by quantitative real-time PCR demonstrated that the levels of *Atrx* transcripts in transgenic oocytes were drastically reduced and represent only 1.1% of the total *Atrx* transcript levels found in non-transgenic controls (Figure 1E and 1F). These results indicate that the *zona pellucida* 3 *(ZP3)-*driven *Atrx*-hairpin vector is a reliable tool for the selective ablation of the ATRX protein during oogenesis.

### Abnormal meiotic chromosome segregation and reduced fertility in transgenic ATRX females

**Figure 2:**
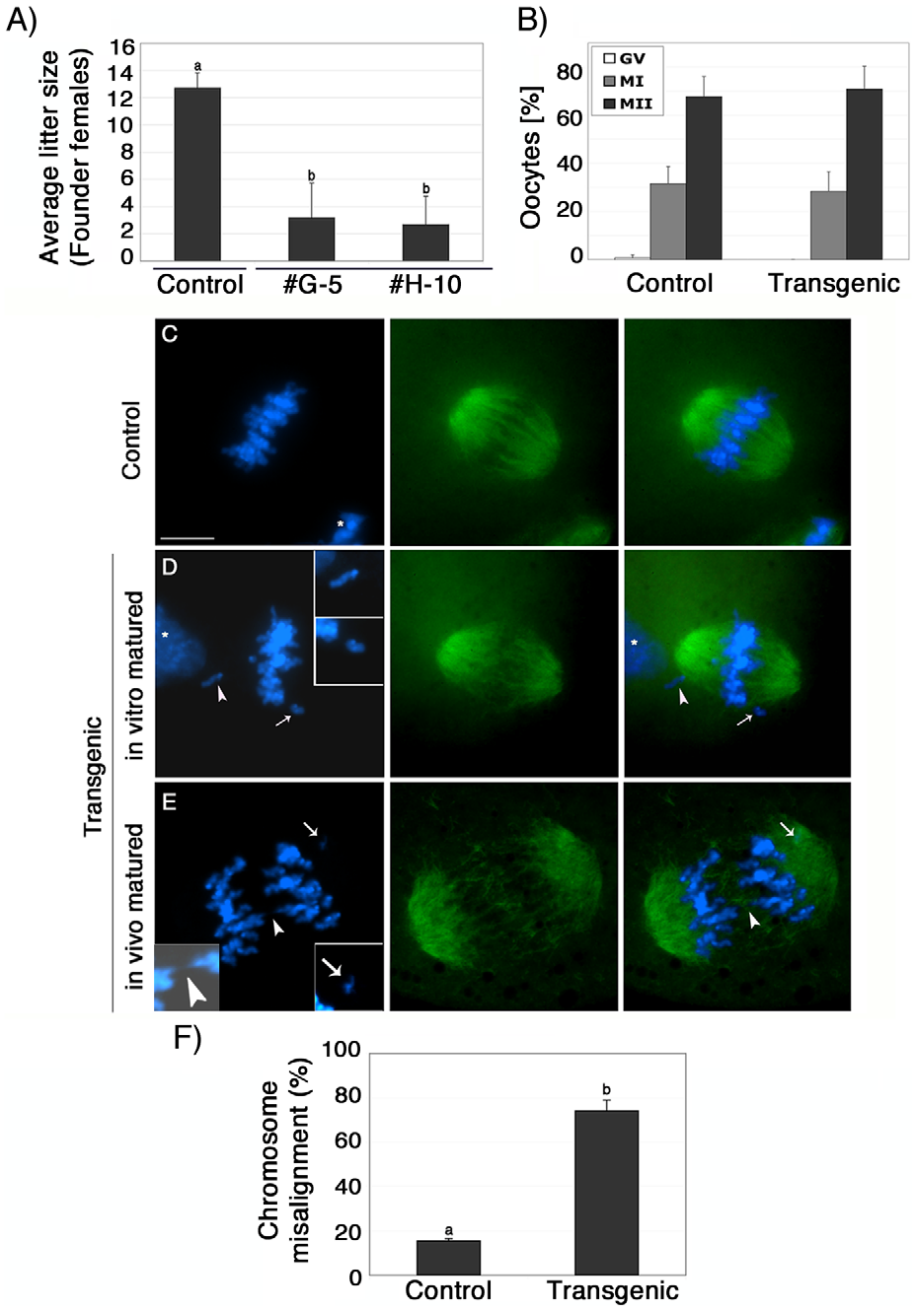
Lack of ATRX function disrupts chromosome alignment at the metaphase II spindle and results in severe sub fertility. (**A**) Control and transgenic female founders were continuously housed with fertile males for a period of 7 months. Transgenic females presented a significant reduction in average litter size (*P*<0.001). (**B**) Proportion of oocytes at the germinal vesicle (GV), metaphase I (MI), or metaphase II (MII) stage following 14 h of *in vitro* maturation. Selective ablation of ATRX has no effect on the progression of meiosis. (**C**) Control *in vitro* matured oocytes show proper chromosome alignment at the metaphase II spindle. In contrast, transgenic oocytes exhibit a spectrum of chromosome segregation defects following maturation *in vitro* and *in vivo* including (**D**) presence of single chromatids (insets) and (**E**) formation of chromosome bridges during premature anaphase II onset (arrowhead, inset shows overexposed DAPI image) as well as lagging chromosomes (arrow and inset). Asterisks indicate the position of the polar body. (**F**) Proportion of *in vitro* matured oocytes with misaligned chromosomes. Data are presented as the mean ± s.d. of three independent experiments. Scale bar  = 10 µm.

To determine whether lack of ATRX function in female gametes influences fertility, we compared the average litter size of transgenic founder females (#G-5 and #H-10) to a wild type littermate control (#F-31). Over a period of 7 months, the transgenic founder female #G-5 produced a total of 6 litters and had an average of 3.2 pups per litter, while founder female #H-10 produced only three litters with an average of 2.7 pups per litter (Figure 2A). In stark contrast, we observed an average litter size of 12.7 pups per litter in a control littermate that produced a total of 7 litters during the observation period. Fertility parameters such as litter size strongly depend on the genetic background of the transgenic strain used [22], [24], [25]. To determine reproductive parameters in ATRX-RNAi females in more detail, we established backcrosses into C57BL/6 as well as outcrosses into the CF1 genetic background (Figure S1E, S1F). As expected, both genetic backgrounds had an effect on the average litter size in both transgenic females as well as controls. However, fertility of transgenic females remained significantly (P<0.05) reduced compared to controls regardless of the particular genetic background used (Figure S1E, S1F).

Analysis of meiotic maturation revealed no significant differences in the proportion of transgenic ova (71%) that reached the metaphase II stage following culture of pre-ovulatory oocytes for 14 h compared with controls (68%), suggesting that functional ablation of ATRX during oogenesis had no effect on meiotic progression (Figure 2B). However, lack of ATRX function during meiosis results in severe chromosome segregation defects in both *in vitro* matured as well as *in vivo* matured oocytes (Figure 2C–2E). For example, compared to *in vitro* matured control oocytes (n = 99) in which chromosomes were tightly aligned at the equatorial region of a bipolar metaphase II spindle (Figure 2C), a high proportion (74.1%; P<0.001) of transgenic oocytes (n = 96) exhibited a range of chromosome segregation defects including lagging chromosomes and loss of sister chromatid cohesion (Figure 2D and 2F). Chromosome segregation defects were also detected in a high proportion of transgenic oocytes by live cell imaging (Figure S3 and Videos S1 and S2). Notably, *in vivo* matured MII oocytes collected from the oviduct of transgenic females, presented a significant increase of chromosomal abnormalities such as misaligned chromosomes, chromosome lagging and in some instances premature anaphase onset with formation of chromosome bridges (Figure 2E, insets) demonstrating that lack of ATRX protein during meiotic maturation *in vivo* and *in vitro* interferes with proper chromosome alignment at the metaphase II stage.

**Figure 3:**
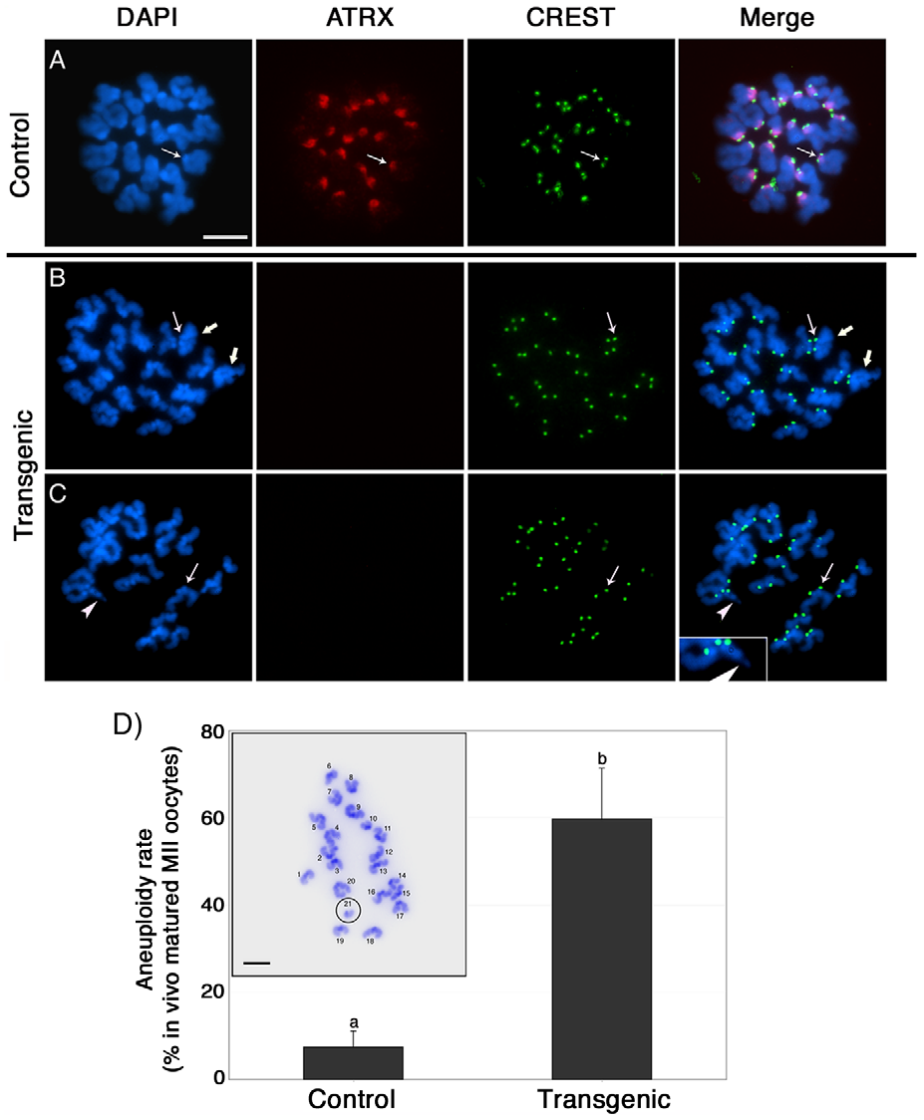
Abnormal chromosome condensation and high incidence of aneuploidy in ATRX deficient oocytes. (**A**) Chromosome spread from a control oocyte at the metaphase II stage showing prominent ATRX staining at pericentric heterochromatin (arrows). (**B**) Lack of ATRX staining at pericentric heterochromatin (thin arrow) is associated with abnormal chromosome condensation (bold arrows) and chromatid breaks (**C**, arrowhead). (**D**) *In vivo* matured transgenic ATRX-RNAi oocytes exhibit a high incidence of aneuploidy. Chromosome spread exhibiting a hyperploid karyotype of 20 chromosomes and an additional single chromatid (circled) is shown. Data are presented as the mean ± s.d. of three independent experiments. Scale bar  = 10 µm.

Next, we determined whether lack of ATRX function during meiosis results in any potential numerical or structural chromosome abnormalities. Analysis of surface spread chromosomes from control oocytes (n = 34) revealed that ATRX (red) associates with a large pericentric heterochromatin domain in contrast with the circumscribed localization of CREST signals to the kinetochore (Figure 3A). As expected, no ATRX protein was detected in transgenic oocytes (n = 35). ATRX-deficient ova exhibit a normal chromosome configuration at the metaphase I stage (Figure S2A). However, at metaphase II lack of ATRX function results in abnormal chromosome morphology associated with incomplete chromosome condensation as well as chromatid breaks in the majority of oocytes (Figure 3B and 3C). At this stage, ATRX deficient oocytes (n = 82) also exhibit a high incidence of aneuploidy (59.7%) compared with only 7.4% observed in controls (n = 66; P<0.001). Cytogenetic analysis of methanol spread chromosomes obtained from *in vivo* matured oocytes revealed that chromosome non-disjunction resulting in hyperploidy (Figure 3D) was the most common type of chromosomal aberrations found in ATRX deficient oocytes, although the presence of single chromatids and premature anaphase II onset was also detected. Taken together, the different types of meiotic spindle and chromosome configuration observed in transgenic oocytes at the metaphase II stage suggest a critical role for ATRX in regulating chromosome segregation and the maintenance of chromosome stability during meiosis. Importantly, our results indicate that lack of ATRX function severely impairs female fertility.

### Functional ablation of ATRX affects the molecular composition of pericentric heterochromatin

**Figure 4:**
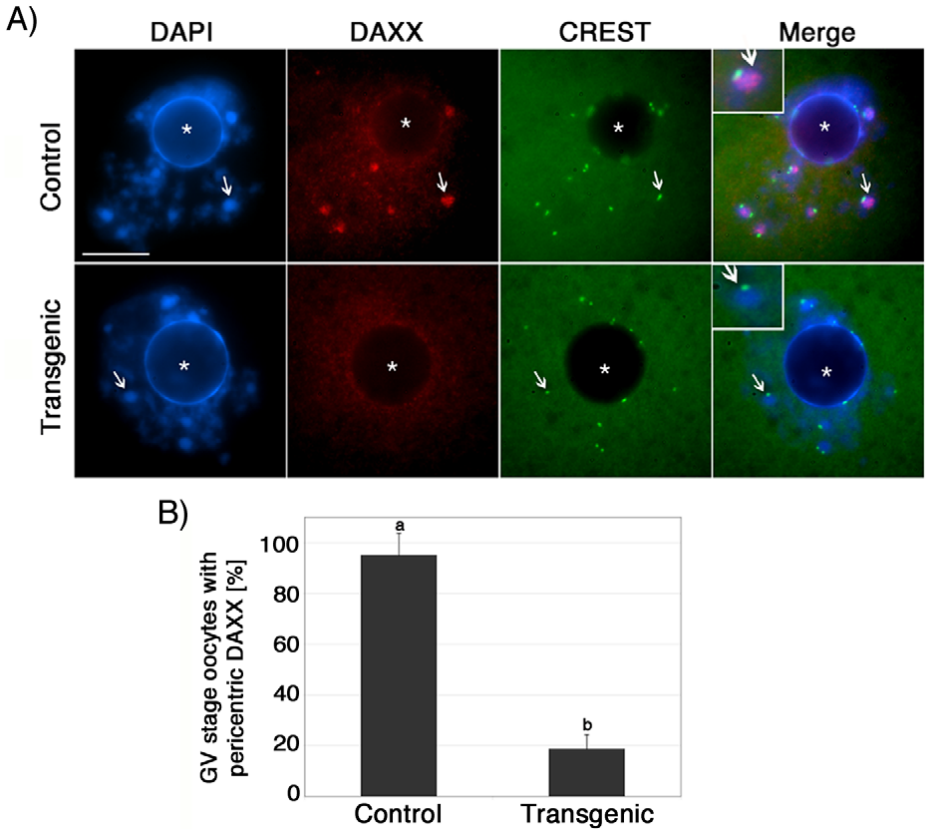
ATRX is required to recruit the transcriptional regulator (DAXX) to pericentric heterochromatin in mammalian oocytes. (**A**) **Top Panel**: Nucleus of a control oocyte showing a precise co-localization of DAXX (red) with bright, DAPI-stained pericentric heterochromatin domains (arrows). The position of the centromere is indicated by CREST (green). **Lower Panel**: Analysis of transgenic oocytes demonstrated that in the absence of ATRX, the transcriptional regulator DAXX fails to associate with pericentric heterochromatin domains while nucleoplasmic expression of DAXX persists. The position of the nucleolus is indicated by (*). (**B**) Proportion of germinal vesicle (GV) stage oocytes showing pericentric DAXX localization. More than 80% of transgenic oocytes fail to recruit DAXX to pericentric heterochromatin. Data are presented as the mean ± s.d. of three independent experiments. CREST immunolocalization (green) was conducted as an experimental control for centromeric-kinetochore integrity. Scale bars  = 10 µm.

ATRX has been shown to exhibit a physical interaction with the transcriptional regulatory factor death domain associated protein (DAXX) at promyelocytic leukemia nuclear bodies (PML's) in both human and murine somatic cells [26]–[28]. However, the mechanisms responsible for recruiting DAXX to pericentric heterochromatin are not known. Therefore we determined whether ATRX is required to recruit DAXX protein to heterochromatin domains in the oocyte genome. Simultaneous analysis of the nuclear localization of DAXX and centromeric proteins detected by the CREST antiserum confirmed that DAXX associates with pericentric heterochromatin domains in control oocytes (n = 74) at the germinal vesicle stage (Figure 4A; arrows). Importantly, analysis of transgenic ova (n = 84) revealed that in the absence of ATRX centromeric signals detected by CREST remain unaffected attesting for kinetochore integrity. However, DAXX protein fails to associate with pericentric heterochromatin domains in 81.3% of germinal vesicle stage oocytes (P<0.005) (Figure 4A and 4B). In contrast, no differences were observed on the patterns of histone H3 trimethylated on lysine 9 (H3K9me3) in ATRX-deficient oocytes compared to controls (Figure S2B) suggesting that this hallmark epigenetic modification is upstream from ATRX binding to constitutive heterochromatin. These results demonstrate for the first time that functional ablation of ATRX affects the molecular composition of pericentric heterochromatin and that ATRX is required to recruit the transcriptional regulator DAXX to these nuclear domains in mammalian oocytes.

### Abnormal chromosome condensation and histone H3 phosphorylation in ATRX deficient oocytes

**Figure 5:**
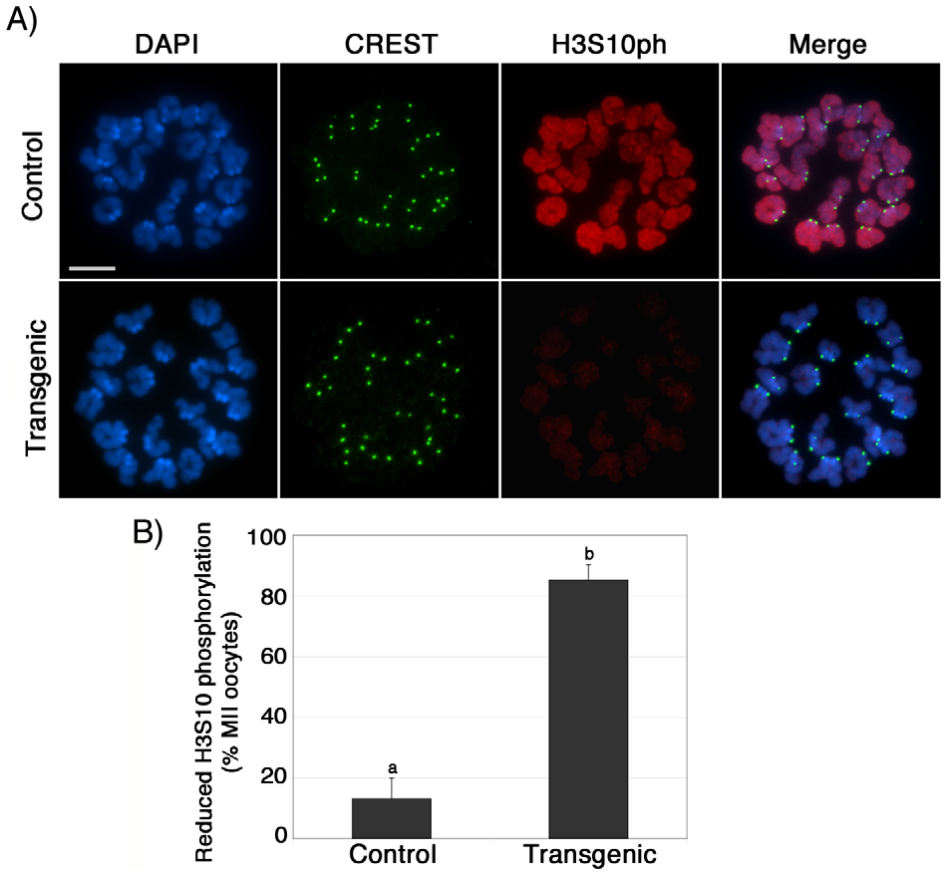
Reduced phosphorylation of histone H3 at serine 10 (H3S10ph) in the chromosomes of ATRX-deficient oocytes. (**A**) Phosphorylation of H3S10 (red) was visualized in metaphase II chromosome spreads from control (upper panel) and transgenic (lower panel) oocytes. Control oocytes exhibit prominent chromosome-wide H3S10ph staining. In contrast, a high proportion of transgenic oocytes exhibit negligible H3S10ph staining at pericentric heterochomatin and throughout the chromatids. CREST immunochemistry (green) served as an experimental control. (**B**) Proportion of oocytes with reduced phosphorylation of H3S10 in transgenic oocytes and controls. Data are presented as the mean ± s.d. of three independent experiments (p<0.05). Scale bar  = 10 µm.

To assess the mechanisms involved in the abnormal chromosome condensation observed in ATRX deficient ova, we determined the patterns of histone H3 phosphorylation at serine 10 (H3S10ph), a chromosome-wide epigenetic modification associated with chromosome condensation [29], [30]. Abnormal H3S10 phosphorylation has been associated with impaired chromosome condensation, chromatid cohesion and aneuploidy [30]–[32]. Notably, a significant proportion (*P*<0.005) of transgenic metaphase II oocytes (85%; n = 73) exhibit reduced levels of H3S10 phosphorylation (red) at both centromeric heterochromatin and along the chromatids when compared to the chromosomes of control oocytes (13% n = 98; Figure 5A and 5B), while immunolocalization of CREST signals (green) at the kinetochores remained essentially unaffected (Figure 5A, lower panel). Consistent with our analysis of transgenic oocytes at the GV stage, lack of ATRX function had no effect on the patterns of H3K9 trimethylation in metaphase II stage oocytes, demonstrating that ATRX knockdown is specifically associated with reduced phosphorylation of H3S10 without affecting the establishment of transcriptionally repressive chromatin modifications such as H3K9me3 (Figure S2C). These results suggest that functional ablation of ATRX might interfere with the establishment and spreading of critical chromatin modifications responsible for proper chromosome condensation and chromatid cohesion during meiosis II.

### Loss of maternal ATRX function results in chromosome instability and the transmission of aneuploidy

**Figure 6:**
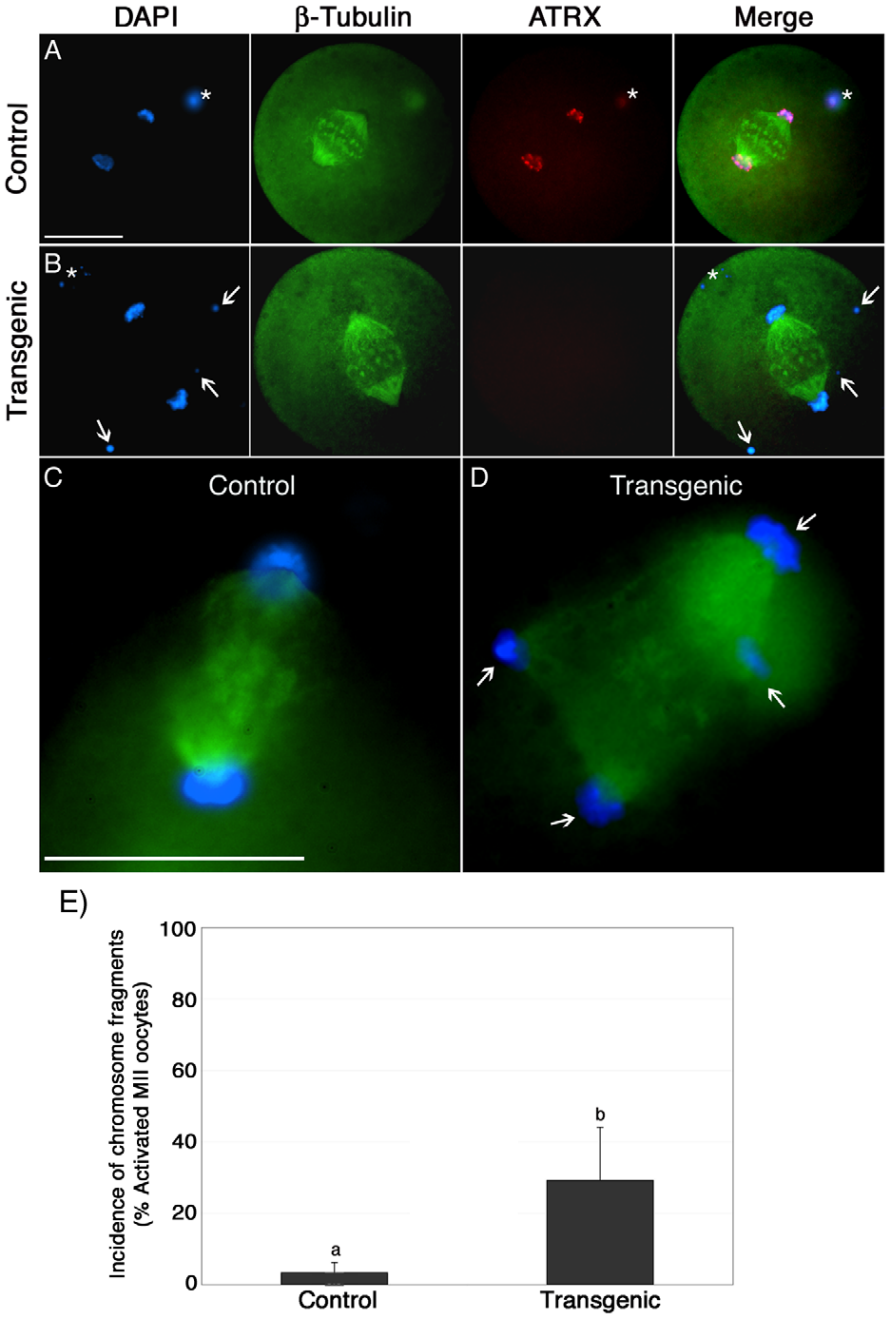
Chromosome fragmentation in ATRX deficient oocytes during the transition to the first mitosis. (**A**) Strontium chloride-activated control and (**B**) transgenic oocytes at late anaphase II stained with ATRX (red) and β-tubulin (green). Numerous chromosomal fragments (arrows) are evident in the transgenic oocyte. Asterisks indicate the position of the polar body. Scale bar  = 50 µm. (**D**) Transgenic oocyte with a multi-polar spindle (arrows, right image) and a control (**C**, left image). Scale bar  = 10 µm. (**E**) Proportion of artificially activated oocytes presenting chromosome fragmentation. Data are presented as the mean ± s.d. of three independent experiments.

To study the dynamics of chromosome segregation during anaphase and determine the type of chromosomal defects transmitted to the embryo, metaphase II stage oocytes were parthenogenetically activated and late anaphase to telophase II oocytes were fixed and processed for immunochemical analysis using antibodies against ATRX (red) and β-tubulin (green, Figure 6). Following parthenogenetic activation, control oocytes (n = 60) showed no evidence of chromosome abnormalities and sister chromatids segregated properly to opposing spindle poles (Figure 6A). However, a significant (*P*<0.05) proportion of transgenic oocytes (29.2%; n = 68) exhibited multiple chromosome fragments (Figure 6B, arrows, and Figure 6E) and, in extreme cases, the formation of multi-polar spindles with dispersion of genetic material throughout the cell (Figure 6D, arrows). This suggests that lack of ATRX function during meiotic progression leads to chromosome instability as defined by the presence of numerical and structural chromosome abnormalities [33] and subsequent chromosome segregation defects during the transition to the first mitosis.

**Figure 7:**
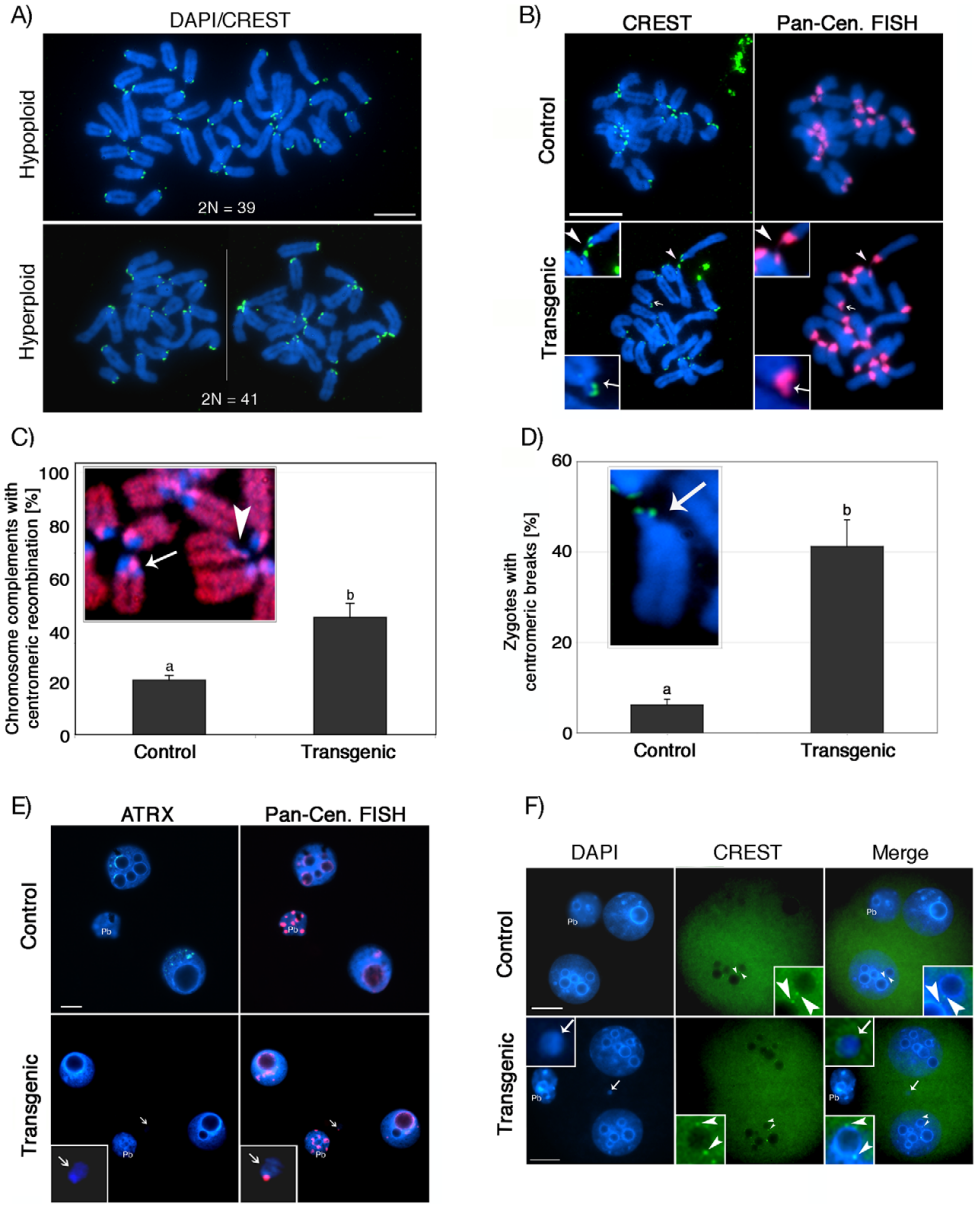
Centromere instability and micronuclei formation following fertilization of ATRX-deficient oocytes. (**A**) Transmission of aneuploidy from the oocyte to the pre-implantation embryo in chromosome spreads obtained from transgenic zygotes analyzed by CREST (green) immunochemistry. Upper panel: transgenic zygote spread with hypoploid karyotype (2N = 39); lower panel: hyperploid karyotype showing 41 chromosomes (micrographs of chromosome complements of this zygote were taken individually (white line) to display at a comparable magnification with the panel above). (**B**) Partial chromosome spreads from zygotes undergoing the first mitotic division. Chromosomes were immunostained with CREST (green) and subsequently subjected to fluorescence *in situ* hybridization using a pan-centromeric DNA probe (red). Transgenic zygotes frequently presented chromosomal breaks at pericentric heterochromatin as indicated by detachment of a chromosomal fragment exhibiting a CREST signal and presence of major satellite DNA (red) at the proximal as well as at the distal fragment (arrow, right inset). Note that satellite DNA sequences in some chromosomes exhibit excessive stretching (arrowhead). (**C**) High incidence (*P*<0.005) of illegitimate centromere mitotic recombination in transgenic parthenogenetic zygotes as indicated by changes in lateral asymmetry of major satellite sequences (arrow). A centromeric break within the same chromosome complement is marked by an arrowhead. (**D**) Proportion of zygotes that exhibit centromeric breaks at the first mitotic division. (**E**) Pan-centromeric FISH revealed that chromosomal instability in transgenic zygotes results in the formation of centromeric DNA-containing micronuclei (arrow, insets) in ATRX deficient 2-cell embryos. (**F**) Distribution of kinetochore domains in 2-cell stage embryos. CREST signals (green) are detectable at perinuleolar regions in the nuclei of control and transgenic embryos (arrowheads, insets). In contrast, micronuclei in transgenic 2-cell stage embryos are CREST-signal negative (arrow, inset), therefore originating from DNA fragmentation and subsequent missegregation of acentric chromosomal material. Scale bars  = 10 µm.

To gain further insight into the potential mechanisms of aneuploidy, we analyzed *in vitro* fertilized zygotes during the first mitotic division by CREST immunochemistry (green). Transmission of aneuploidy was clearly evident in transgenic zygotes as indicated by the presence of numerical chromosome abnormalities (Figure 7A). Moreover, immuno-FISH experiments with CREST antiserum (green) and subsequent visualization of pericentric heterochromatin using a pan-centromeric DNA probe (red) revealed the presence of centromeric breaks (Figure 7B lower panel, arrow and inset) in a high proportion of *in vitro* fertilized transgenic zygotes (41.2%; n = 105) compared to controls (6.6%; n = 100) (*P*<0.001; Figure 7-D). Moreover, we observed extensive stretching of centromeric heterochromatin domains in transgenic zygotes (Figure 7-B, lower panel, arrowhead), indicating that chromosome breaks and structural aberrations occur within constitutive heterochromatin. This notion was further substantiated by a significant increase in the incidence (44.7%; n = 65) of illegitimate recombination events within centromeric heterochromatin in parthenogenetic ATRX-RNAi zygotes compared to controls (20.9%; n = 85), which was reflected by the presence of sister chromatid exchanges (red) during the first DNA replication cycle (*P*<0.005; Figure 7C, arrow). Interestingly, we also found evidence for the presence of centromeric breaks within the same chromosomal spreads (arrowhead), which might give rise to acentric chromosomal fragments and the formation of micronuclei. Notably, compared to controls (5.6%; n = 90), a higher proportion of *in vitro* fertilized transgenic 2-cell embryos (39.6%; n = 102) exhibited micronuclei (Figure 7E, lower panel, arrow and inset). Moreover, virtually all micronuclei observed at this stage contained major satellite DNA corresponding to pericentric heterochromatin (red) along with euchromatic chromosomal fragments (Figure 7E). However, in contrast to blastomere nuclei (arrowheads, inset), kinetochore domains (CREST, green) were not detectable in micronuclei of transgenic 2-cell embryos (Figure 7-F, arrow, inset) demonstrating that most micronuclei originate from acentric chromosomal fragments rather than whole chromosomes. These findings provide strong evidence that lack of ATRX results in the transmission of aneuploidy and the occurrence of centromeric breaks leading to a high incidence of structural and numerical chromosome aberrations in the pre-implantation embryo.

**Figure 8:**
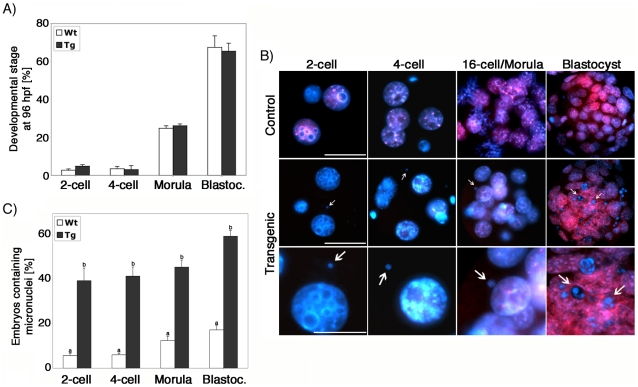
Lack of ATRX function during oogenesis results in severe genomic instability in the pre-implantation embryo. (**A**) Developmental potential of ATRX deficient oocytes. Control and transgenic oocytes exhibit similar rates of blastocyst formation at 96 hours following *in vitro* fertilization (hpf). (**B,C**) Presence of micronuclei throughout pre-implantation development in ATRX deficient embryos. Although ATRX protein can be detected at the 8–16 cell stage following fertilization of ATRX deficient oocytes, a high proportion of ATRX deficient embryos exhibit micronuclei formation (arrow) as evidence of chromosome instability at all stages evaluated. Data are presented as the mean ± s.d. of three independent experiments. Scale bar  = 10 µm.

Surprisingly, despite the formation of micronuclei and centromeric instability, progression to the blastocyst stage was similar for transgenic and control embryos (Figure 8A). Immunostaining of control pre-implantation embryos using antibodies against ATRX (red) revealed a characteristic localization pattern to DAPI-bright heterochromatic domains (Figure 8B, upper panel). In transgenic embryos, however, ATRX protein became gradually detectable beginning at the 8–16 cell stage, presumably as a result of zygotic gene activation and a cessation of the RNAi effect (Figure 8B, middle panel). Interestingly, micronuclei formation in transgenic embryos was prevalent throughout pre-implantation development and increased from 39.6% (n = 67) at the 2-cell stage to 59.8% (n = 83) at the blastocyst stage (Figure 8B lower panel, arrows). Controls demonstrated micronuclei in significantly lower proportions at all developmental stages 5.7% (n = 71) at the 2-cell stage to 17.3% (n = 88) in blastocysts, respectively, (Figure 8C), demonstrating that lack of ATRX in the female gamete results in severe chromosomal instability throughout pre-implantation development.

## Discussion

The underlying mechanisms that predispose the female gamete to a high incidence of aneuploidy during meiosis are not well defined. In this study, we provide evidence that maintenance of pericentric heterochromatin structure and function is essential for chromosome stability in the female germ line. Our findings indicate that the chromatin remodeling protein ATRX plays a critical role in heterochromatin formation and chromosome segregation in the mammalian oocyte. Lack of ATRX function during oocyte growth results in severe sub-fertility and has a profound effect on the molecular composition of pericentric heterochromatin as demonstrated by failure to recruit the transcriptional regulator DAXX to the chromocenters of transgenic pre-ovulatory oocytes. At the metaphase II stage, ATRX-deficient oocytes exhibit a high incidence of aneuploidy due to chromosome non-disjunction, presence of single chromatids as well as premature anaphase II onset. Notably, abnormal segregation was also associated with aberrant chromosome morphology, reduced phosphorylation of histone H3 at serine 10 (H3S10ph) and centromeric breaks, suggesting a role for ATRX in chromosome condensation-related events and centromere stability during meiosis. Collectively, our results revealed a critical pathway in which ATRX is required for recruitment of DAXX to pericentric heterochromatin in the mammalian oocyte genome. Moreover, our study indicates that the role of ATRX in chromosome segregation might be mediated through an epigenetic mechanism involving the maintenance of chromatin modifications associated with pericentric heterochromatin compaction and chromosome condensation. Importantly, the type of chromosome segregation defects found during meiotic progression of transgenic oocytes *in vivo* and *in vitro* are consistent with a role for ATRX in maintaining chromosome stability at metaphase II and during the transition to the first mitosis.

In human and murine somatic cells, interaction of the ATRX protein with DAXX results in the formation of a novel transcriptionally repressive complex with chromatin remodeling activity [26]–[28]. In HeLa cells, the transcriptional regulator DAXX establishes a physical interaction with ATRX where it plays an active role in recruiting ATRX to promyelocytic leukemia bodies (PML's) [26]–[28]. In turn, DAXX accumulation at heterochromatic domains is associated with phosphorylation of ATRX [26]. However, the mechanisms responsible for recruiting DAXX to pericentric heterochromatin remained to be established. We now provide direct evidence that ATRX is required for the recruitment of DAXX to pericentric heterochromatin domains in the female germ line. The presence of normal histone trimethylation (H3K9me3) in transgenic oocytes indicates that ATRX is downstream from this repressive mark but upstream from DAXX and hence provide novel insight on a critical pathway of heterochromatin assembly in the oocyte genome. Therefore, by recruiting transcriptional regulatory factors, ATRX plays a critical role in pericentric heterochromatin formation and in regulating the molecular composition of constitutive heterochromatin domains during oocyte growth. Notably, in stark contrast with somatic cells in which the association of DAXX with ATRX at pericentric heterochromatin is restricted to a brief period at the S phase of the cell cycle [26], [28], DAXX protein remains associated with ATRX at pericentric heterochromatin in the nucleus of fully-grown oocytes. Thus, in female germ cells the interaction between DAXX and ATRX is maintained beyond the S phase as mammalian dictyate oocytes are arrested at a stage equivalent to the G2/M transition, a process that might be of functional significance for the establishment of unique chromatin remodeling events in the oocyte genome.

The functional consequences of ATRX ablation on centromere structure and function are not fully understood. However, our results indicate that at least one chromatin modification, phosphorylation of histone H3 at serine 10 (H3S10ph), which is known to initiate at pericentric heterochromatin at the G2/M transition and subsequently distributes throughout the chromatids following chromosome condensation [29], [34], [35], is dramatically reduced in ATRX deficient oocytes and therefore may contribute to the abnormal chromosome condensation observed in transgenic oocytes at the metaphase II stage. The underlying epigenetic mechanisms involved in this process are currently under investigation. However, recent studies indicate that H3S10ph is severely affected by factors known to disrupt DNA methylation in somatic cells [36]. Although ATRX does not contain a canonical DNA methyltransferase motif, DNMT3B and ATRX share a closely related plant homeodomain (PHD)-like zinc finger domain [37], [38] and patients with ATRX syndrome exhibit alterations in DNA methylation at repetitive sequences [39]. Moreover, recent studies indicate that spontaneous mutations occurring at the PHD domain of several chromatin remodeling factors, including ATRX, may severely affect their ability to interpret specific epigenetic marks resulting in striking changes in chromatin structure [40]. Therefore, it is conceivable that lack of ATRX function might interfere with chromosome-wide H3S10 phosphorylation by inducing either a conformational change in chromatin configuration or an abnormal DNA methylation pattern at pericentric heterochromatin. In turn, H3S10ph is governed by members of the aurora kinase family, which are essential for the formation of a bipolar spindle and the recruitment of the condensin complex in order to ensure sister chromatid cohesion [31]. Hence, chromosomal defects in conjunction with global reduction of H3S10 phosphorylation in ATRX deficient oocytes suggest that pericentric ATRX might be critical for chromosome condensation and sister chromatid cohesion. Our studies indicate that abnormal chromatin modifications might contribute to chromosome instability in ATRX deficient oocytes and thus constitute a valuable model to unravel the potential involvement of abnormal chromatin modifications on the onset of aneuploidy in the female gamete.

Consistent with our previous studies [12], ATRX-deficient oocytes progress to the metaphase II stage, however, while chromosomes at metaphase I appear normal, more than 70% of transgenic oocytes at the metaphase II stage exhibit chromosome segregation defects including lagging chromosomes and to a lesser extent, premature separation of sister chromatids, suggesting a role for ATRX in chromosome segregation and sister chromatid cohesion. Chromosome congression defects have also been observed following transient depletion of ATRX in human somatic cells [41], indicating that ATRX is important for proper chromosome segregation during mitotic as well as meiotic cell division in mammals. However, the higher frequency of chromosome non-disjunction observed in ATRX deficient oocytes (our study) compared to somatic cells (30%) [41] might reflect subtle albeit important differences in ATRX function during meiosis and hence further studies are required to determine the molecular mechanisms predisposing ATRX deficient oocytes to a high incidence of chromosomal non-disjunction.

Both cytogenetic analyses as well as live cell imaging studies indicate that chromosome segregation defects in ATRX deficient oocytes arise during the metaphase II stage. For example, the presence of a single misaligned chromosome might reflect a specific disruption of kinetochore-microtubule interactions underscoring a potential ‘crosstalk’ between centromeric and pericentric heterochromatin in the epigenetic control of centromere function in mammalian oocytes. Interestingly, this process may not be sufficient to trigger the spindle assembly checkpoint in ATRX deficient oocytes as evidenced by the lack of metaphase I arrest. However, it confers the oocyte with the potential to transmit aneuploidy to the early conceptus. On the other hand, the presence of single chromatids and premature anaphase II onset in transgenic oocytes matured *in vivo* suggest a potential role for ATRX in centromeric cohesion. Consistent with this notion, studies in human and murine somatic cells have recently demonstrated a role for ATRX in centromeric cohesion [41]. Interestingly, abnormal regulation of *Atrx* transcripts has also been observed in cohesin deficient cell lines obtained from patients with Cornelia de Lange Syndrome (CdLs) [42].

The presence of chromosome fragments containing major satellite DNA sequences during the transition to the first mitosis is indicative of centromere instability in the absence of ATRX and might result from microtubule tension during the transition to the first mitosis. Micronuclei formation is a prominent indicator of extensive chromosomal damage and identification of the type of micronuclei prevalent provides critical insight into the underlying mechanisms of aneuploidy. For example, micronuclei might correspond to small acentric fragments resulting from double strand break formation or, alternatively, to whole chromosomes induced by abnormal segregation during previous cell divisions [43]. We demonstrate here, that ATRX-depleted oocytes and embryos contain both numerical chromosome aberrations as well as centromeric break-associated micronuclei reflecting severe chromosome instability. In addition, pericentric heterochromatin of transgenic zygotes shows an increased rate of mitotic recombination events. Tandem repeats in centromeric satellite DNA are thought to form hot-spots for illicit sister chromatid exchanges requiring repressive heterochromatin marks to prevent changes in centromeric repeat sequence length and hence impairment of centromere function. For instance, increased rates of centromere mitotic recombination have previously been correlated with DNA hypomethylation in ES cells lacking the methyltransferases Dnmt3a and Dnmt3b underscoring the significance of epigenetic marks in the repression of mitotic sister chromatid exchanges [44]. In conclusion, our data identify ATRX as an essential epigenetic component of pericentric heterochromatin for the repression of centromere mitotic rearrangements, DNA breaks and chromosome missegregation and hence as an important guardian of centromere integrity and function.

Chromosome segregation errors during meiosis lead to aneuploidy in approximately 10–25% of all human conceptions and human embryos are particularly susceptible to aneuploidy, which in the majority of cases is inherited through the female gamete [45], [46]. Although the underlying mechanisms that promote a high incidence of aneuploidy are not fully understood, advanced maternal age is widely recognized as the single most important etiological factor for an increased risk of chromosomal non-disjunction and premature separation of sister chromatids [47], [48]. The two mechanisms of aneuploidy most commonly observed in ATRX deficient oocytes, namely non-disjunction of whole chromosomes and premature separation of sister chromatids are also the major causes of aneuploidy in oocytes from women of advanced age [49]. Therefore ATRX deficient ova constitute an invaluable model to determine the molecular mechanisms involved in the onset of aneuploidy as a function of maternal age. Interestingly, transcriptome analyses conducted in oocytes from young and aged female mice revealed a significant reduction of *Atrx* mRNA levels [50], [51] consistent with the notion that loss of ATRX function during reproductive senescence may contribute to the onset of aneuploidy in the female gamete. The reduced fertility observed in ATRX knockdown female mice underscores the importance of this model to determine the molecular mechanisms of aneuploidy and its effects on female fertility.

## Materials and Methods

### ATRX-RNAi construct design and generation of transgenic animals

All animal experiments were approved by the institutional animal use and care committee of the University of Pennsylvania according to National Institutes of Health guidelines. A two-step cloning strategy was used to direct the insertion of an *Atrx* inverted repeat into a vector driving the expression of the enhanced green fluorescent protein (EGFP) under the control of a *zona pellucida*-3 gene promoter (pZP3-intron-EGFP), a generous gift from Dr. R. Schultz [21], [52]. Briefly, the *Atrx* coding sequence spanning exon 3 to exon 5 was amplified from mouse oocyte cDNA using the *BglII* and *XbaI* restriction site-containing primer pair “Atrx-fwd1” 5′-aga tct gga aaa taa caa gga aga ggg agc-3′ and “Atrx rev” 5′-tct aga acg cag tca cca agt cca gta gag-3′. A shorter fragment was amplified using the primer pair “Atrx-fwd2” 5′-gga tcc cag agc cag tgc tga atg aag ac-3′ (*BamHI*) and “Atrx rev”. Both PCR products were individually sub-cloned into vector pCR4-TOPO (Invitrogen, Carlsbad, CA) before *NotI/BglII*-excision of the longer hairpin fragment and ligation into a *BamHI/NotI*-linearized pCR4-TOPO vector carrying the shorter fragment. Following *XbaI* digest, the inverted repeat was inserted into vector *pZP3*-intron-EGFP to generate *pZP3-intron-EGFP-Atrx-hp* (Figure S1A). Microinjection of the construct into pronuclear stage embryos was conducted at the University of Pennsylvania's Transgenic Mouse Facility. Transgenic mice were identified by PCR using the primer pair “EGFPseq-F” 5′-aaa gac ccc aac gag aag cg-3′ and ”Atrx-fwd2” or by presence of a 301 bp amplicon originating from the *SV40* intron as described previously [21]. Transgenic lines were established through outcrossing of transgenic founders with CF1 wild type mice. To test the fertility of transgenic founders (C57BL/6/SJL/J), females were mated with CF1 males at a ratio of 1∶1 and housed together for a period of 7 months. CF1-outcrossed female offspring (B6SJL/CF1) as well as C57BL/6-backcrossed female offspring (n = 3–4/line) and appropriate wild-type controls (n = 6 for each experiment) were mated with C57BL/6/SJL/J males at approximately 40 days of age and continuously housed at a ratio of 1∶1 for a period of 7 months. Litter size was recorded and is displayed as the average per transgenic line or control group.

### Oocyte and embryo culture, analysis of centromeric sister chromatid exchanges, and chromosome spreading

Fully-grown GV stage oocytes were recovered from 22–24 day old females 48 h post injection with 5 IU of pregnant mare serum gonadotropin (PMSG; National Hormone and Peptide Program, NIDDK). Oocytes were collected in MEM medium supplemented with 3 mg/ml bovine serum albumin (MEM/BSA; Sigma Aldrich, Inc. St. Louis, MO), and 10 µM Milrinone (Sigma) to prevent germinal vesicle breakdown. *In vitro* matured metaphase II oocytes were obtained following culture in fresh MEM/BSA media supplemented with 5% fetal bovine serum (FBS, Hyclone, Logan, UT) for 14 h under an atmosphere of 5% O_2_, 5% CO_2_ and 90% N_2_ at 37°C. *In vivo* matured metaphase II oocytes were collected from the oviducts of superovulated females 14 h post human chorionic gonadotropin injection (5 IU; EMD Biosciences, Inc. La Jolla, CA). Early zygotes and pre-implantation stage embryos were obtained following *in vitro* fertilization and culture of *in vivo* derived eggs in KSOM medium as described [53]. *In vivo* matured metaphase II oocytes were parthenogenetically activated as described previously [54] and late anaphase/telophase II oocytes were fixed for chromosome and spindle analysis. Chromosomal spreads from cleavage stage embryos were obtained following exposure to 100 ng/ml colchicine for 4 h (GIBCO, Life Technologies, Grand Island NY). Karyotyping was conducted on surface spread chromosomes following treatment with methanol/acetic acid (MeOH/AA) essentially as described [55]. Centromeric sister chromatid exchanges were detected through changes in the lateral asymmetry in the C-band region of chromosomes as revealed by incorporation of BrdU (5-bromo-2′-deoxyuridine) during a single replication cycle [56]. Lateral asymmetry is thought to reflect an unequal distribution of thymidine residues between adjacent C-band regions of sister chromatids resulting in a strongly asymmetric fluorescent signal following incorporation and detection of BrdU nucleotides. These regions of asymmetrical brightness have been shown to consist of centromeric satellite sequences by chromosome orientation fluorescence *in situ* hybridization (CO-FISH) [56], [57] and hence allow the visualization and quantification of centromere mitotic recombination events [44]. To analyze the incidence of centromeric sister chromatid exchanges in the ATRX-RNAi model, parthenogenetic transgenic and control zygotes were cultured in 250 µM BrdU from 6 to 24 h post activation (hpa). In addition, colchicine (100 ng/ml) was added to the culture media at 12 hpa to arrest chromosomes at the first mitotic division before surface spreading of chromosomes complements onto glass slides.

### Immunochemistry

Ovaries from transgenic females and control (wild type) littermates (26 days of age) were dissected and fixed in Bouin's solution before processing for paraffin sectioning (5 µm) and staining with hematoxylin and eosin according to standard procedures. For whole mount immunochemistry, maturing oocytes and pre-implantation embryos were washed in MEM/BSA and fixed in 4% paraformaldehyde (PFA) and 0.1% Triton X 100 for 20 minutes at 37°C. Analysis of chromosomal proteins was conducted on surface spread chromosomes prepared following exposure of zona-free oocytes to a solution of 1% PFA, 0.1% Triton X 100 as described [58] and subsequently blocked with PBS containing 1 mg/ml BSA and 0.01% Triton X 100 for 1 h at room temperature or overnight at 4°C. Polyclonal rabbit anti-ATRX and anti-DAXX antibodies were obtained from Santa Cruz Biotechnology, Inc. (Santa Cruz, CA) and used at a dilution of 1∶200. Mouse anti-γ-tubulin was used at 1∶6000 and mouse anti-β-tubulin at 1∶400 (Sigma). Human anti-CREST antiserum was a generous gift of Dr. W. Earnshaw and used at a concentration of 1∶5000. Mouse anti-H3S10ph (Histone 3 phosphorylated at serine 10, 1∶1000) and rabbit anti-H3K9me3 (Histone 3 trimethylated at lysine 9, 1∶200) were purchased from Millipore (Billerica, MA, USA) and Abcam Inc. (Cambridge, MA, USA), respectively. Incorporation of BrdU nucleotides was detected as previously described [16]. Immunodetection was performed using appropriate Alexa Fluor-conjugated secondary antibodies (Molecular Probes, Eugene, Oregon, USA) at a dilution of 1∶1000 for 2 h at room temperature. Samples were counterstained and mounted onto glass slides in mounting medium containing DAPI (4′, 6-diamidino-2-phenylindole; Vecta Shield plus DAPI, Vector Laboratories, Inc. Burlingame, CA).

### RNA isolation and quantitative RT-PCR

Messenger RNA was isolated from denuded oocytes using Micro-Fast Track 2.0 kit (Invitrogen) and subsequently subjected to reverse transcription using oligo-dT primer and the Superscript II first strand synthesis system (Invitrogen). The resulting cDNA was used for quantitative expression analysis of *Atrx* transcripts by real-time PCR using FastStart DNA master SYBR green I kit (Roche, Indianapolis, IN) on a Roche Light Cycler apparatus. Primer sequences were as follows: “Atrx-RT-Fwd” 5′-ctg cct tca cac act gga ttt tg-3′ and “Atrx-RT-Rev” 5′-cgg agt tca cca tca tct gct g-3′, corresponding to sequences within exon 9 and exon 11, respectively. Real-time PCR results were normalized against β-actin transcript levels as a housekeeping control (primer pair: “β-actin-Fwd” 5′-gat atc gct gcg ctg gtc gtc-3′; “β-actin-Rev” 5′-acg cag ctc att gta gaa ggt gtg g-3′).

### Live cell imaging

Time-lapse image acquisition was performed following microinjection of capped messenger RNA encoding a histone H2B-GFP fusion protein in fully-grown GV stage oocytes to visualize chromosomes as described [59]. Microinjected oocytes were cultured in the presence of 10 µM milrinone for 3–4 h to allow recombinant protein expression before *in vitro* maturation for 16 h in an oil-covered micro drop of M2 medium maintained at 37°C using an incubation chamber. Germinal vesicle breakdown and polar body extrusion was monitored by 2D time-lapse microscopy at 20 minutes intervals on a Leica TCS-SP5 laser scanning confocal microscope equipped with a 10x objective lens and 2.37x digital zoom. Chromosome segregation was analyzed by 3D time-lapse imaging following excitation of GFP protein with a 488 nm argon laser and detected using 505–550 nm emission filter. Live cell imaging data were analyzed by 3D reconstructions using LAS AF software (Leica, Bannockburn, IL).

### Fluorescence *in situ* hybridization (DNA-FISH)

Following immunochemistry, slides were processed for DNA-FISH analysis using a Cy3-conjugated Pan-centromeric probe (Cambio Ltd., Cambridge, UK), according to manufacturer's specifications and with the following modifications. Briefly, surface spread interphase nuclei and metaphase chromosomes were denatured in 70% formamide (VWR International Ltd., Poole, UK) in 2X SSC at 85°C for 10 minutes and subsequently chilled in ice-cold 70% ethanol for 5 minutes. The pan-centromeric probe was denatured for 10 minutes at 85°C and incubated at 37°C for 1 h. Overnight hybridization was carried out in a humidified chamber at 37°C and stringency washes were conducted in a solution containing 50% formamide in 2X SSC as previously described [60].

### Statistical analysis

Data presented as percentage values were analyzed by one-way analysis of variance (ANOVA) following arcsine transformation. Comparison of all pairs was conducted by the Tukey-Kramer HSD or Student's t-test using JMP Start Statistics (SAS Institute Inc., Cary, NC). Variation among individual replicates is indicated as the standard deviation (s.d.). Differences were considered significant when *P*<0.05 and are indicated by different superscripts.

### Image acquisition

Immunofluorescence and FISH image acquisition of paraformaldehyde or methanol/acetic acid fixed samples was performed at room temperature using Vectashield (Vector Laboratories) as mounting medium. Data analysis was conducted using a Leica DMRE fluorescence microscope (Leica Microsystems, Inc.) equipped with a HCX PLAN APO 40x/0.85 air, and with a PLAN APO 63x/1.20 water objective. Images were captured with a Leica DFC 350F camera using Openlab 3.1.7. software (PerkinElmer) and image processing was performed using Photoshop 2.0 (Adobe) for linear adjustments and cropping of fluorescent images. No gamma adjustments were made.

## Supporting Information

Figure S1 Strategy to generate an oocyte-specific knockdown of ATRX using a transgenic RNAi approach. (A) DNA construct used to generate ATRX-RNAi transgenic mice. The transgene consists of the Zona pellucida protein 3 (ZP3) promoter sequence, an SV40 intron, enhanced green fluorescent protein (EGFP) coding sequence, an Atrx-specific inverted repeat and an SV40 early polyadenylation signal. The nucleotide positions of Atrx cDNA sequence forming the inverted repeat are indicated. Arrows depict the position of primer pairs used for genotyping. (B) Transgenic founder animals exhibiting germ line transmission and penetrance of the ATRX knockdown. (C) Ovarian sections obtained from wild type control and transgenic females 26 days of age showing a similar number of antral/preovulatory follicles (arrows) and pre-antral follicles (arrowheads), suggesting that transgenic oocytes develop normally up to the pre-ovulatory stage. (D) The proportion of oocytes with ATRX staining (black bars) at pericentric heterochromatin was dramatically reduced in all transgenic lines generated. (E) Average litter size on different transgenic lines in the CF1 out-crossed transgenic progeny and first-generation female offspring of a C57BL/6 backcross (F). Different superscripts indicate significant differences (p<0.05).
(4.08 MB TIF)

Figure S2 Nuclear and chromosome configuration in ATRX deficient oocytes at different stages of meiosis. (A) Chromosome spreads of control and transgenic metaphase I oocytes following 8 h of in vitro maturation showing proper chromosome condensation and centromere cohesion (arrow) in ATRX deficient ova. (B) Histone H3 trimethylated at lysine 9 (H3K9me3, red) co-localizes with pericentric heterochromatin (arrow) and the perinucleolar heterochromatin rim (arrowhead) in control and transgenic oocytes at the germinal vesicle stage. (C) Similarly, H3K9me3 remains localized at pericentric domains in ATRX-deficient oocytes. CREST immunolocalization (green) served as experimental control. Scale bars  = 10 µm.
(4.18 MB TIF)

Figure S3 Live cell imaging of meiotic maturation in ATRX deficient oocytes. Z-stack reconstructions of laser scanning confocal micrographs obtained by live cell imaging at indicated time points during in vitro maturation of H2B-GFP expressing control and transgenic ATRX-RNAi oocytes.
(1.23 MB TIF)

Video S1 Time lapse control oocyte. Time lapse (interval  = 20 minutes) movie of laser scanning confocal Z-stack reconstructions (every 1.3 µm) of H2B-GFP expressing control oocytes during in vitro maturation.
(0.65 MB MOV)

Video S2 Time lapse transgenic oocyte. Time lapse (interval  = 20 minutes) movie of laser scanning confocal Z-stack reconstructions (every 1.3 µm) of H2B-GFP expressing transgenic oocytes during in vitro maturation.
(0.61 MB MOV)

## References

[1] Festenstein R, Sharghi-Namini S, Fox M, Roderick K, Tolaini M (1999). Heterochromatin protein 1 modifies mammalian PEV in a dose- and chromosomal-context-dependent manner.. Nat Genet.

[2] Bernard P, Maure J, Partridge J, Genier S, Javerzat J (2001). Requirement of heterochromatin for cohesion at centromeres.. Science.

[3] Bernard P, Allshire R (2002). Centromeres become unstuck without heterochromatin.. Trends Cell Biol.

[4] Dillon N, Festenstein R (2002). Unravelling heterochromatin: competition between positive and negative factors regulates accessibility.. Trends Genet.

[5] Cleveland D, Mao Y, Sullivan K (2003). Centromeres and kinetochores: from epigenetics to mitotic checkpoint signaling.. Cell.

[6] Craig JM, Earle E, Canham P, Wong LH, Anderson M (2003). Analysis of mammalian proteins involved in chromatin modification reveals new metaphase centromeric proteins and distinct chromosomal distribution patterns.. Hum Mol Genet.

[7] Guenatri M, Bailly D, Maison C, Almouzni G (2004). Mouse centric and pericentric satellite repeats form distinct functional heterochromatin.. J Cell Biol.

[8] Sullivan BA, Karpen GH (2004). Centromeric chromatin exhibits a histone modification pattern that is distinct from both euchromatin and heterochromatin.. Nat Struct Mol Biol.

[9] Karpen GH, Allshire RC (1997). The case for epigenetic effects on centromere identity and function.. Trends Genet.

[10] Maison C, Almouzni G (2004). HP1 and the dynamics of heterochromatin maintenance.. Nat Rev Mol Cell Biol.

[11] McDowell TL, Gibbons RJ, Sutherland H, O'Rourke DM, Bickmore WA (1999). Localization of a putative transcriptional regulator (ATRX) at pericentromeric heterochromatin and the short arms of acrocentric chromosomes.. Proc Natl Acad Sci USA.

[12] De La Fuente R, Viveiros M, Wigglesworth K, Eppig J (2004). ATRX, a member of the SNF2 family of helicase/ATPases, is required for chromosome alignment and meiotic spindle organization in metaphase II stage mouse oocytes.. Developmental Biology.

[13] Hakimi M, Bochar D, Schmiesing J, Dong Y, Barak O (2002). A chromatin remodelling complex that loads cohesin onto human chromosomes.. Nature.

[14] Peters AH, O'Carroll D, Scherthan H, Mechtler K, Sauer S (2001). Loss of the Suv39h histone methyltransferases impairs mammalian heterochromatin and genome stability.. Cell.

[15] Maison C, Bailly D, Peters A, Quivy J, Roche D (2002). Higher-order structure in pericentric heterochromatin involves a distinct pattern of histone modification and RNA component.. Nature Genetics.

[16] Baumann C, De La Fuente R (2009). ATRX marks the inactive X chromosome (Xi) in somatic cells and during imprinted X chromosome inactivation in trophoblast stem cells.. Chromosoma.

[17] Baumann C, Schmidtmann A, Muegge K, De La Fuente R (2008). Association of ATRX with pericentric heterochromatin and the Y chromosome of neonatal mouse spermatogonia.. BMC Molecular Biology.

[18] Ritchie K, Seah C, Moulin J, Isaac C, Dick F (2008). Loss of ATRX leads to chromosome cohesion and congression defects.. J Cell Biol.

[19] Page S, Hawley R (2003). Chromosome choreography: the meiotic ballet.. Science.

[20] Petronczki M, Siomos M, Nasmyth K (2003). Un menage a quatre: the molecular biology of chromosome segregation in meiosis.. Cell;.

[21] Stein P, Svoboda P, Schultz RM (2003). Transgenic RNAi in mouse oocytes: a simple and fast approach to study gene function.. Dev Biol;.

[22] Ma J, Zeng F, Schultz R, Tseng H (2006). Basonuclin: a novel mammalian maternal-effect gene.. Development.

[23] Fedoriw A, Stein P, Svoboda P, Schultz R, Bartolomei M (2004). Transgenic RNAi reveals essential function for CTCF in H19 gene imprinting.. Science.

[24] Mayer A, Bulian D, Scherb H, Hrabé de Angelis M, Schmidt J (2007). Emergency prevention of extinction of a transgenic allele in a less-fertile transgenic mouse line by crossing with an inbred or outbred mouse strain coupled with assisted reproductive technologies.. Reprod Fertil Dev.

[25] Silver LM (1995). Mouse genetics.. Concepts and applications.

[26] Ishov AM, Vladimirova OV, Maul GG (2004). Heterochromatin and ND10 are cell-cycle regulated and phosphorylation-dependent alternate nuclear sites of the transcription repressor Daxx and SWI/SNF protein ATRX.. J Cell Sci.

[27] Tang J, Wu S, Liu H, Stratt R, Barak OG (2004). A novel transcription regulatory complex containing death domain-associated protein and the ATR-X syndrome protein.. J Biol Chem.

[28] Xue Y, Gibbons R, Yan Z, Yang D, McDowell TL (2003). The ATRX syndrome protein forms a chromatin-remodeling complex with Daxx and localizes in promyelocytic leukemia nuclear bodies.. Proc Natl Acad Sci USA.

[29] Hendzel MJ, Wei Y, Mancini MA, Van Hooser A, Ranalli T (1997). Mitosis-specific phosphorylation of histone H3 initiates primarily within pericentromeric heterochromatin during G2 and spreads in an ordered fashion coincident with mitotic chromosome condensation.. Chromosoma.

[30] Wei Y, Yu L, Bowen J, Gorovsky M, Allis C (1999). Phosphorylation of histone H3 is required for proper chromosome condensation and segregation.. Cell.

[31] Giet R, Glover D (2001). Drosophila aurora B kinase is required for histone H3 phosphorylation and condensin recruitment during chromosome condensation and to organize the central spindle during cytokinesis.. J Cell Biol.

[32] Swain JE, Ding J, Brautigan DL, Villa-Moruzzi E, Smith GD (2007). Proper chromatin condensation and maintenance of histone H3 phosphorylation during mouse oocyte meiosis requires protein phosphatase activity.. J Biol Reprod.

[33] Foijer F, Draviam VM, Sorger PK (2008). Studying chromosome instability in the mouse.. Biochim Biophys Acta.

[34] Cobb J, Miyaike M, Kikuchi A, Handel MA (1999). Meiotic events at the centromeric heterochromatin: histone H3 phosphorylation, topoisomerase II alpha localization and chromosome condensation.. Chromosoma.

[35] Zeitlin S, Barber C, Allis C, Sullivan K (2001). Differential regulation of CENP-A and histone H3 phosphorylation in G2/M.. J Cell Sci.

[36] Monier K, Mouradian S, Sullivan KF (2007). DNA methylation promotes Aurora-B-driven phosphorylation of histone H3 in chromosomal subdomains.. J Cell Sci.

[37] Okano MBD, Haber DA, Li E (1999). DNA methyltransferases Dnmt3a and Dnmt3b are essential for de novo methylation and mammalian development.. Cell.

[38] Xie S, Wang Z, Okano M, Nogami M, Li Y (1999). Cloning, expression and chromosome locations of the human DNMT3 gene family.. Gene.

[39] Gibbons RJ, McDowell TL, Raman S, O'Rourke DM, Garrick D (2000). Mutations in ATRX, encoding a SWI/SNF-like protein, cause diverse changes in the pattern of DNA methylation.. Nat Genet.

[40] Baker LA, Allis CD, Wang GG (2008). PHD fingers in human diseases: Disorders arising from misinterpreting epigenetic marks.. Mutation Research.

[41] Ritchie K, Seah C, Moulin J, Issac C, Dick F (2008). Loss of ATRX leads to chromosome cohesion and congression defects.. J Cell Biol.

[42] Liu J, Zhang Z, Bando M, Itoh T, Deardorff MA (2009). Transcriptional dysregulation in NIPBL and cohesin mutant human cells.. PLoS Biol.

[43] Iarmarcovai G, Botta A, Orsiere T (2006). Number of centromeric signals in micronuclei and mechanisms of aneuploidy.. Toxicology Letters.

[44] Jaco I, Canela A, Vera E, Blasco MA (2008). Centromere mitotic recombination in mammalian cells.. J Cell Biol.

[45] Hassold T, Hunt P (2001). To err (meiotically) is human: The genesis of human aneuploidy.. Nature Reviews Genetics.

[46] Hunt PA, Hassold TJ (2008). Human female meiosis: what makes a good egg go bad?. Trends Genet.

[47] Eichenlaub-Ritter U (2005). Mouse genetic models for aneuploidy induction in germ cells.. Cytogenet Genome Res.

[48] Lamb NE, Sherman SL, Hassold TJ (2005). Effect of meiotic recombination on the production of aneuploid gametes in humans.. Cytogenet Genome Res.

[49] Vialard F, Petit C, Bergere M, Gomes DM, Martel-Petit V (2006). Evidence of a high proportion of premature unbalanced separation of sister chromatids in the first polar bodies of women of advanced age.. Hum Reprod.

[50] Hamatani T, Falco G, Carter MG, Akutsu H, Stagg CA (2004). Age-associated alteration of gene expression patterns in mouse oocytes.. Hum Mol Genet.

[51] Pan H, Ma P, Zhu W, Schultz RM (2008). Age-associated increase in aneuploidy and changes in gene expression in mouse eggs.. Developmental Biology.

[52] Svoboda P, Stein P, Schultz RM (2001). RNAi in mouse oocytes and preimplantation embryos: effectiveness of hairpin dsRNA.. Biochem Biophys Res Commun.

[53] De La Fuente R, O'Brien MJ, Eppig JJ (1999). Epidermal growth factor enhances preimplantation developmental competence of maturing mouse oocytes.. Hum Reprod.

[54] Boiani M, Eckardt S, Leu N, Schöler H, McLaughlin K (2003). Pluripotency deficit in clones overcome by clone-clone aggregation: epigenetic complementation?. EMBO J.

[55] Tarkowski A (1966). An air-drying method for chromosome preparations from mouse eggs.. Cytogenetics.

[56] Goodwin EH, Meyne J, Bailey SM, Quigley D (1996). On the origin of lateral asymmetry.. Chromosoma.

[57] Falconer E, Chavez EA, Henderson A, Poon SSS, McKinney S (2010). Identification of sister chromatids by DNA template strand sequences.. Nature.

[58] Yang F, Baumann C, De La Fuente R (2009). Persistence of histone H2AX phosphorylation after meiotic chromosome synapsis and abnormal centromere cohesion in poly (ADP-ribose) polymerase (Parp-1) null oocytes.. Dev Biol.

[59] Igarashi H, Knott JG, Schultz RM, Williams CJ (2007). Alterations of PLCβ1 in mouse eggs change calcium oscillatory behavior following fertilization.. Developmental Biology.

[60] De La Fuente R, Viveiros M, Burns K, Adashi E, Matzuk M (2004). Major chromatin remodeling in the germinal vesicle (GV) of mammalian oocytes is dispensable for global transcriptional silencing but required for centromeric heterochromatin function.. Dev Biol.

