# Supplementary figures and images for: Loss of Maternal ATRX Results in Centromere Instability and Aneuploidy in the Mammalian Oocyte and Pre-Implantation Embryo
Source: PLoS Genet. 2010 Sep 23;6(9):e1001137. doi: 10.1371/journal.pgen.1001137 (PMC2944790; doi:10.1371/journal.pgen.1001137)

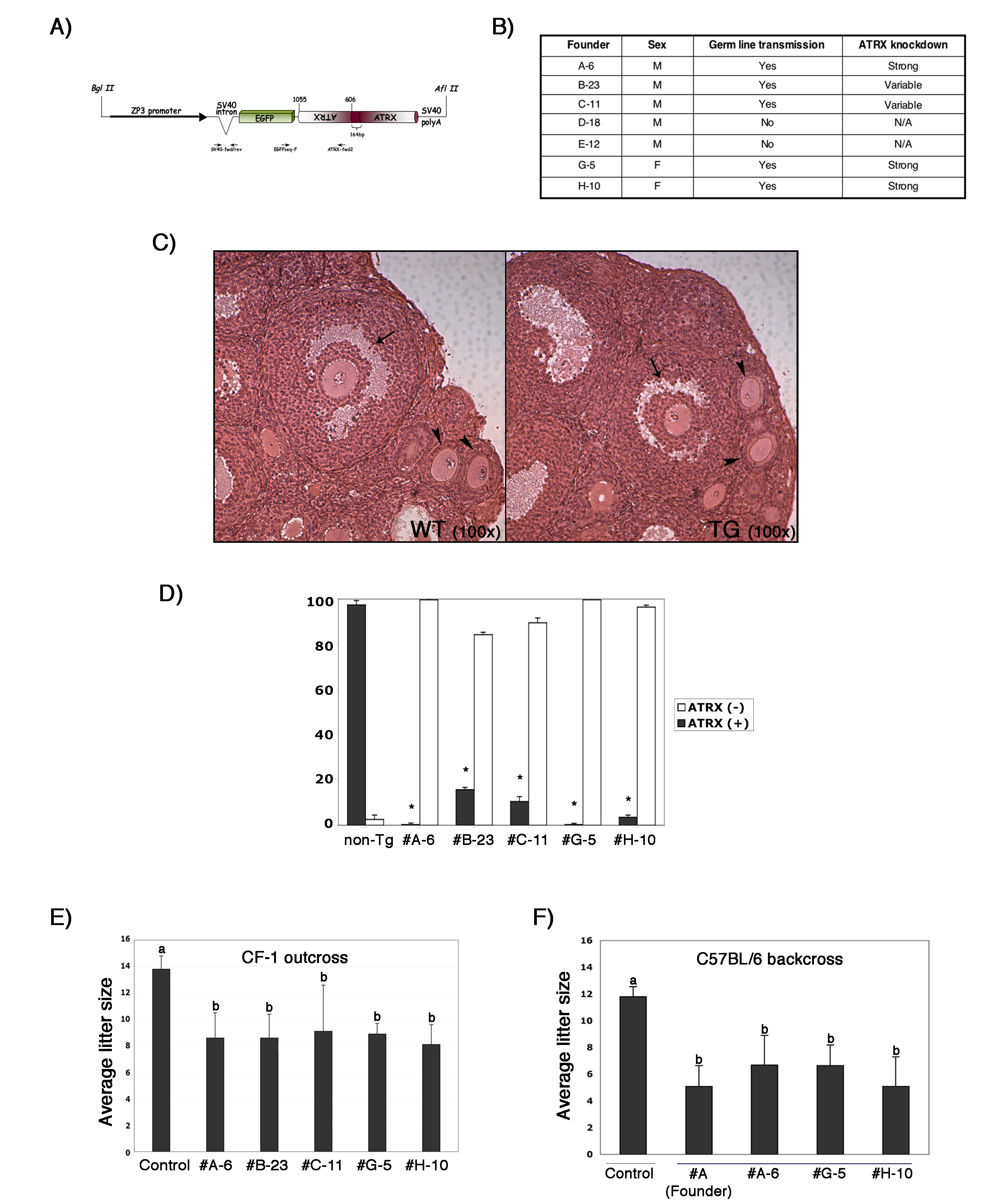

Supplement: Figure S1 — Strategy to generate an oocyte-specific knockdown of ATRX using a transgenic RNAi approach. (A) DNA construct used to generate ATRX-RNAi transgenic mice. The transgene consists of the Zona pellucida protein 3 (ZP3) promoter sequence, an SV40 intron, enhanced green fluorescent protein (EGFP) coding sequence, an Atrx-specific inverted repeat and an SV40 early polyadenylation signal. The nucleotide positions of Atrx cDNA sequence forming the inverted repeat are indicated. Arrows depict the position of primer pairs used for genotyping. (B) Transgenic founder animals exhibiting germ line transmission and penetrance of the ATRX knockdown. (C) Ovarian sections obtained from wild type control and transgenic females 26 days of age showing a similar number of antral/preovulatory follicles (arrows) and pre-antral follicles (arrowheads), suggesting that transgenic oocytes develop normally up to the pre-ovulatory stage. (D) The proportion of oocytes with ATRX staining (black bars) at pericentric heterochromatin was dramatically reduced in all transgenic lines generated. (E) Average litter size on different transgenic lines in the CF1 out-crossed transgenic progeny and first-generation female offspring of a C57BL/6 backcross (F). Different superscripts indicate significant differences (p<0.05). (4.08 MB TIF) [file pgen.1001137.s001.tif]

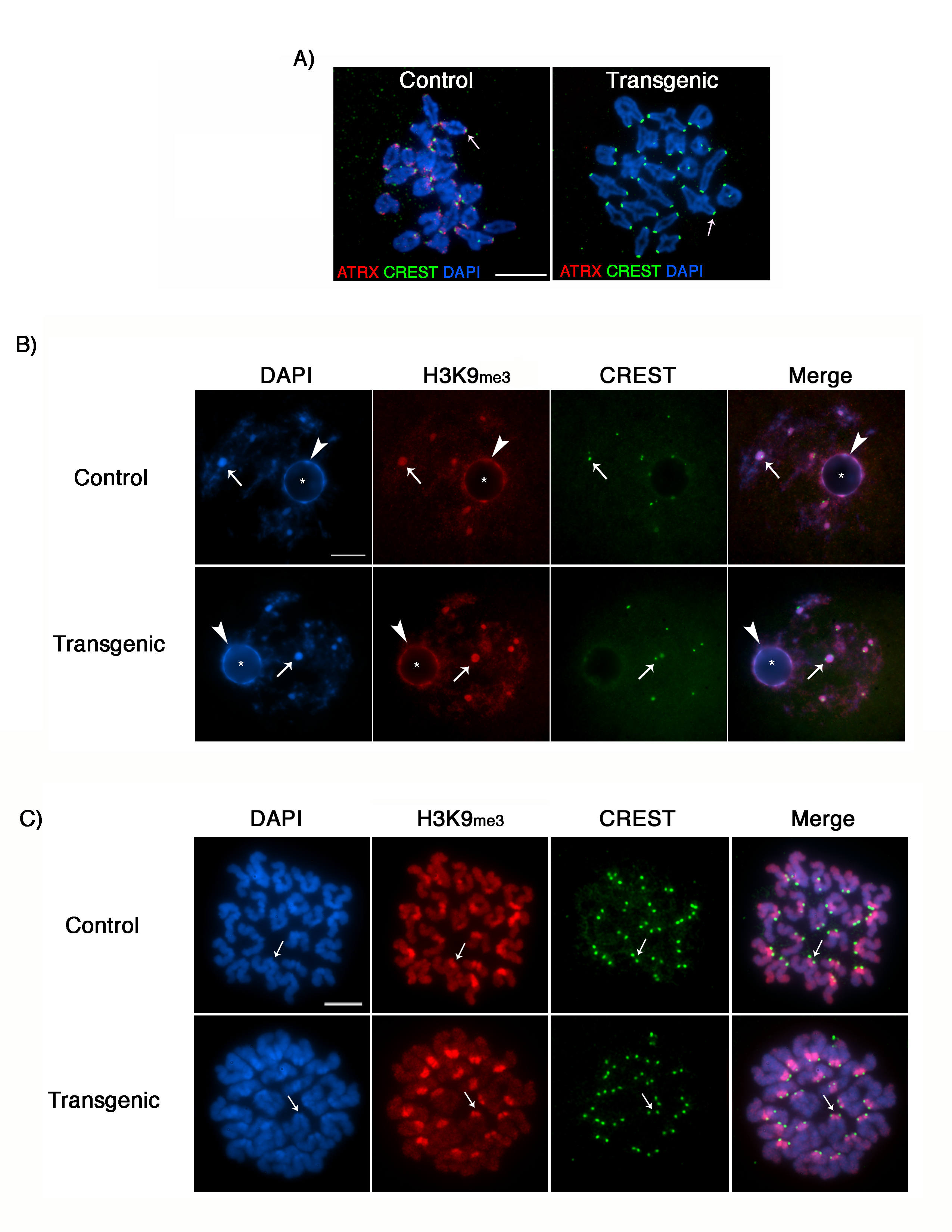

Supplement: Figure S2 — Nuclear and chromosome configuration in ATRX deficient oocytes at different stages of meiosis. (A) Chromosome spreads of control and transgenic metaphase I oocytes following 8 h of in vitro maturation showing proper chromosome condensation and centromere cohesion (arrow) in ATRX deficient ova. (B) Histone H3 trimethylated at lysine 9 (H3K9me3, red) co-localizes with pericentric heterochromatin (arrow) and the perinucleolar heterochromatin rim (arrowhead) in control and transgenic oocytes at the germinal vesicle stage. (C) Similarly, H3K9me3 remains localized at pericentric domains in ATRX-deficient oocytes. CREST immunolocalization (green) served as experimental control. Scale bars = 10 µm. (4.18 MB TIF) [file pgen.1001137.s002.tif]

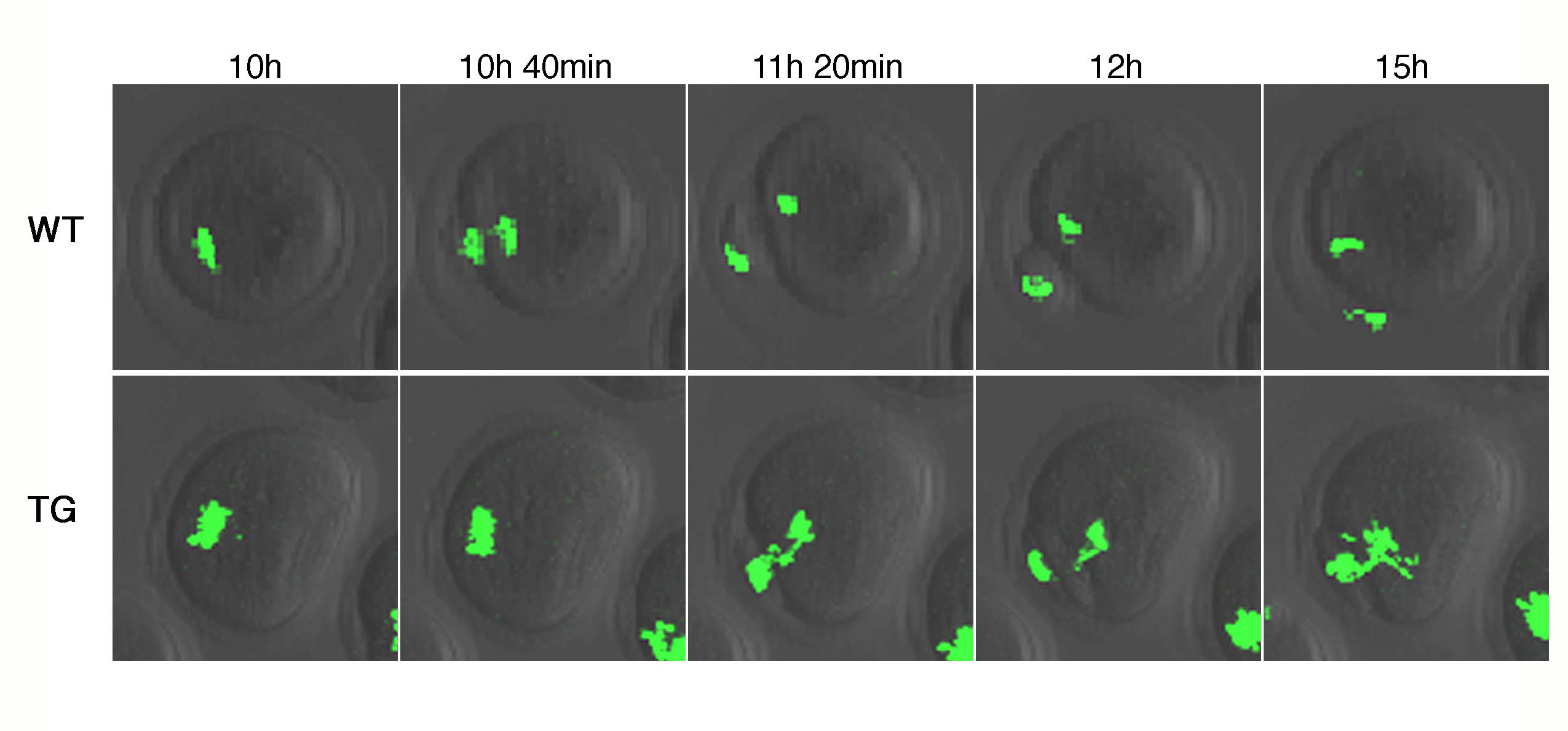

Supplement: Figure S3 — Live cell imaging of meiotic maturation in ATRX deficient oocytes. Z-stack reconstructions of laser scanning confocal micrographs obtained by live cell imaging at indicated time points during in vitro maturation of H2B-GFP expressing control and transgenic ATRX-RNAi oocytes. (1.23 MB TIF) [file pgen.1001137.s003.tif]
